# A Scoping Review of the Impact of Environmental Design on Wayfinding for People With Sensory Impairment

**DOI:** 10.1177/19375867251391361

**Published:** 2025-11-20

**Authors:** Parastoo Zali, Lori B. McElroy, Mario Ettore Giardini, Kullapat Chaiyawat, Margaret Watson

**Affiliations:** 1 Department of Architecture, 3527 University of Strathclyde, Glasgow, UK; 2 Department of Biomedical Engineering, 3527 University of Strathclyde, Glasgow, UK; 3 School of Science and Engineering, 3042 University of Dundee, Dundee, UK; 4 Strathclyde Institute of Pharmacy and Biomedical Sciences, 14306 University of Strathclyde, Glasgow, UK

**Keywords:** spatial navigation, architectural accessibility, wayfinding, visual impairment, hearing impairment

## Abstract

**Purpose:**

This review aimed to identify the environmental factors impacting wayfinding by people with sensory impairment (SI) and the perceived barriers and facilitators of those factors. In addition, the review explored design recommendations to improve the accessibility of built environments for this population.

**Background:**

Wayfinding design is frequently misconceived as the implementation of signage, whereas it also involves spatial planning to facilitate intuitive navigation. Individuals with visual and hearing impairments face multiple accessibility challenges that could be tackled through user-centered design.

**Methods:**

A scoping review was conducted using standard methodology. Electronic databases were searched (Medline, Embase, APA PsycINFO, SCOPUS, Web of Science) from January 2000 to August 2023. Independent duplicate screening was performed for 10% of sources. The extracted data was analyzed using content analysis. A conceptual framework was developed to map the key environmental factors impacting the individual's wayfinding with SI.

**Results:**

From the 3,716 records identified, 41 studies were included. Results were categorized into three domains of architectural, graphical, and sensory elements. Frequently cited architectural barriers included complex layouts, unclear circulation, nonstandard stairs, and the presence of obstacles. Regarding graphical elements, the nonstandard design or placement of signage was common. Key sensory challenges were related to insufficient lighting, low visual contrast, and the inappropriate selection of materials.

**Conclusions:**

This review highlighted multiple environmental factors that influence wayfinding for people with SI. Policymakers, architects, and designers could use these results to eliminate barriers in the built environment and develop evidence-based design interventions addressing the access needs of this population.

## Introduction

Sensory impairment (SI), particularly visual and hearing impairments, affects many people worldwide, and this is exacerbated by the global rise in aging populations. Globally, around 2.2 billion people have some form of visual impairment (VI) (WHO, 2022), and hearing impairment (HI) affects more than 1.5 billion people (one in five people) (WHO, 2023). Since both senses are crucial for effective wayfinding, their impairment or loss can significantly hinder a person's capacity for spatial orientation and localization (Xiong et al., 2023). Reduced mobility and orientation negatively impact quality of life, especially for older adults with SI (Tseng et al., 2018). This places additional strain on caregivers, and the existing infrastructure becomes unsuitable when designed without accessibility in mind (Beach et al., 2023).

*Navigation*, *spatial orientation*, and *wayfinding* are related concepts often used interchangeably. Navigation refers to traveling along a chosen route (Karimi, 2015) to reach a destination. Spatial orientation is one's ability to determine their location in a setting (Arthur & Passini, 1992, p. 23), focusing on the cognitive processes of understanding spatial layout. This article adopts the definition developed by Arthur and Passini (1992) that wayfinding is “spatial problem-solving” encompassing the interrelated processes of decision-making, decision execution, and information processing. Wayfinding performance is typically assessed using a combination of objective and subjective indicators, including efficiency (time taken, travel distance, and speed rate), accuracy (reaching the intended destination and the number of errors), as well as behavioral and cognitive measures (O’Neill, 1992; Ruddle & Lessels, 2006).

Wayfinding design involves two dimensions of spatial planning and environmental communication (through visual, auditory, and tactile cues). This is also linked to the concept of cognitive mapping, which involves forming a mental image of a spatial layout. Lynch (1960) identified five key elements—regions, edges, nodes, paths, and landmarks—that people use to construct a cognitive map of a city. Wayfinding design is of growing importance in the domain of architectural and urban design. Previous literature reviews have studied the accessibility needs of people with SI (Chidiac et al., 2024; Seetharaman et al., 2024), but their main focus was the outdoor environment. Jamshidi et al. (2020) explored the relationship between indoor wayfinding and environmental factors; however, they excluded individuals with impairments in this study. A gap remains in the holistic understanding of the dynamics between the wayfinding behavior of people with SI and environmental design. Addressing this knowledge gap could inform architects and designers to design spaces that promote greater independence and accessibility for people living with SI alongside other users.

*A gap remains in the holistic understanding of the dynamics between the wayfinding behavior of people with SI and environmental design.*

## Review Aims and Objectives

The scoping review aimed to map scholarly evidence and identify gaps related to: (a) the environmental factors affecting navigation and wayfinding for individuals with SI and (b) design recommendations that promote accessibility of built environments for this population.

## Methods

The scoping review protocol reflects the current JBI (Joanna Briggs’ Institute) methodology (Pollock et al., 2023) and the Preferred Reporting Items for Systematic Reviews and Meta-Analyses extension for Scoping Reviews (PRISMA-ScR) guidelines (Tricco et al., 2018). The protocol was registered on the Open Science Framework on June 19, 2023 (Zali et al., 2023).

### Eligibility Criteria

The inclusion criteria were developed based on the Population, Concept, Context (PCC) framework (Peters et al., 2020). Studies were included if they involved people living with visual, hearing, or dual impairment (Population), addressed the barriers, facilitators, and design strategies for navigation and wayfinding (Concept) within the built environment, including both indoors and outdoors (Context). Studies about wayfinding technologies not applicable to the built environment (such as wearable assistive devices, smart canes, and smartphone apps) were excluded. Empirical studies using any study design were included. Studies were excluded if they reported secondary data, e.g., reviews. Studies were included only if the full text was available. No geographical or language restrictions were applied. The initial protocol stated that grey literature would be included. Due to the volume of empirical data identified, grey literature was not included in this review.

### Search Strategy

The search strategy was constructed using a combination of keywords, MeSH (Medical Subject Heading) terms, and Boolean operators related to the review objectives and tailored to each database. The strategy was peer-reviewed by experienced librarians. Five bibliographic databases were searched from January 2000 to August 2023, including Medline [Ovid], Embase [Ovid], APA PsycINFO, SCOPUS, and Web of Science [Core Collection]. The search strategy used for Medline (Ovid) is provided in the Supplementary Materials.

### Selection of Sources of Evidence

Search results were imported into EndNote 20 (Clarivate Analytics, PA, USA) reference management software and then imported into CADIMA (JKI - Julius Kühn-Institut, n.d.), to remove duplicates and assist the screening process. Non-English papers were translated using Google Translate before screening. One reviewer (PZ) conducted a three-stage screening (title, abstract, full text) for all sources. At each stage, another reviewer (KC) performed independent duplicate screening of 10% of sources. Disagreements were resolved through discussions. Reasons for exclusion were recorded at the full-text stage.

### Data Extraction and Critical Appraisal

A data extraction form was developed in Microsoft Excel, including the citation details of evidence, elements of the PCC framework, study design, main findings, and findings related to each review question. This form was shared and discussed with the team during the extraction process to ensure it was accurate and captured the required information. When the extraction form was modified, the data extraction was repeated for all sources so that the final version had up-to-date data. During the data extraction, authors were contacted for missing data and/or for clarification. Critical appraisal was conducted to assess the risk of bias and credibility of the results using the Mixed Methods Appraisal Tool (MMAT) (Hong, Pluye et al., 2018).

### Data Analysis

**Figure 1.**
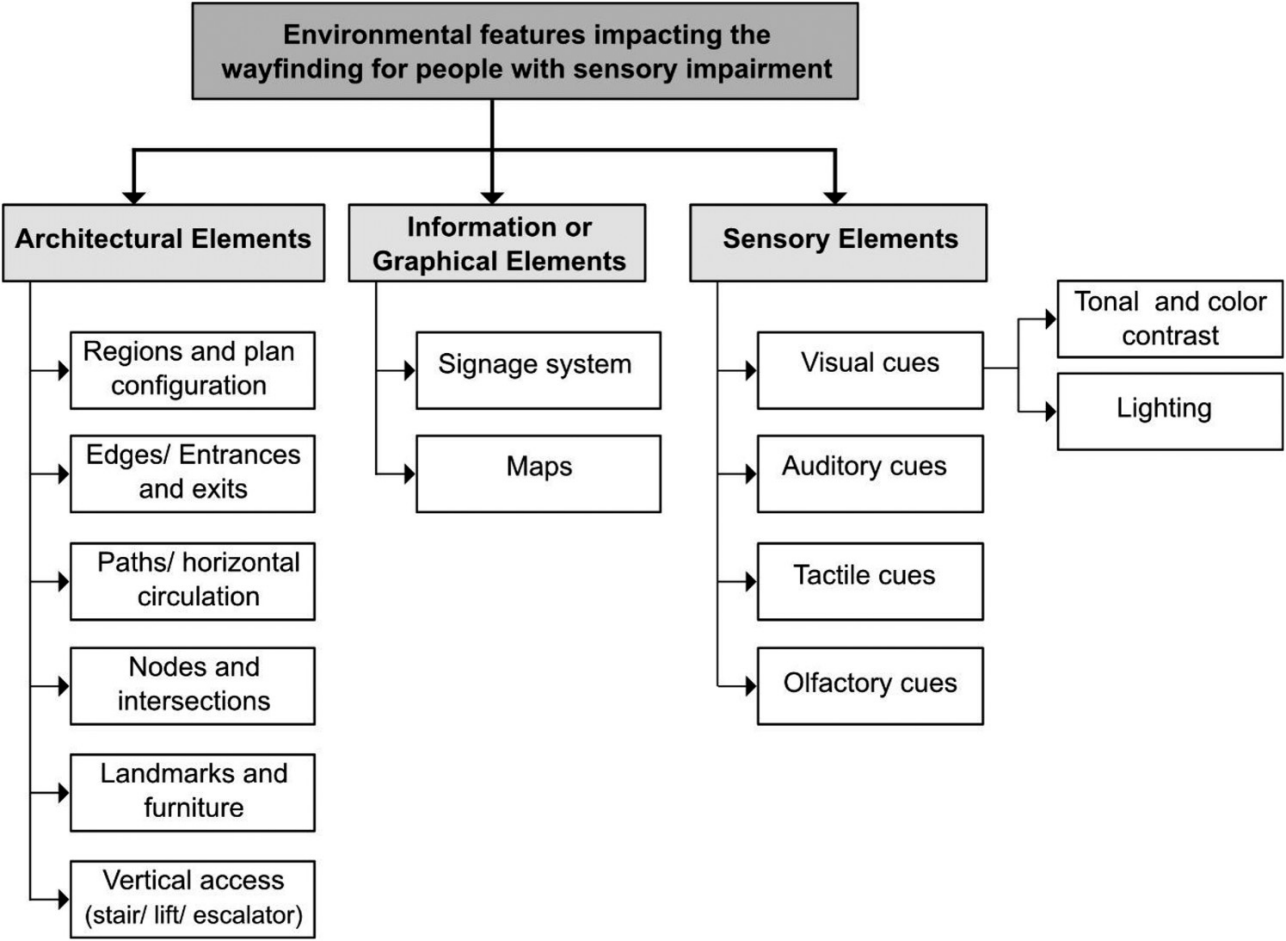
The Conceptual Framework Illustrating Key Environmental Features That Impact Wayfinding for People With Sensory Impairment.

Content analysis was undertaken to address the review objectives using inductive and deductive approaches. A conceptual framework was developed to map the extracted data. The process started with reviewing the evidence sources and open coding of the extracted data to identify potential categories. An initial coding structure was developed in response to review objectives. Theories and models of wayfinding were used to inform the framework, including Arthur and Passini's (1992) classification for wayfinding design and Lynch's (1960) environmental factors in wayfinding (regions, edges, nodes, paths, and landmarks). The framework was finalized by combining the results of the two stages and re-mapping the extracted data (Figure 1).

## Results

**Figure 2.**
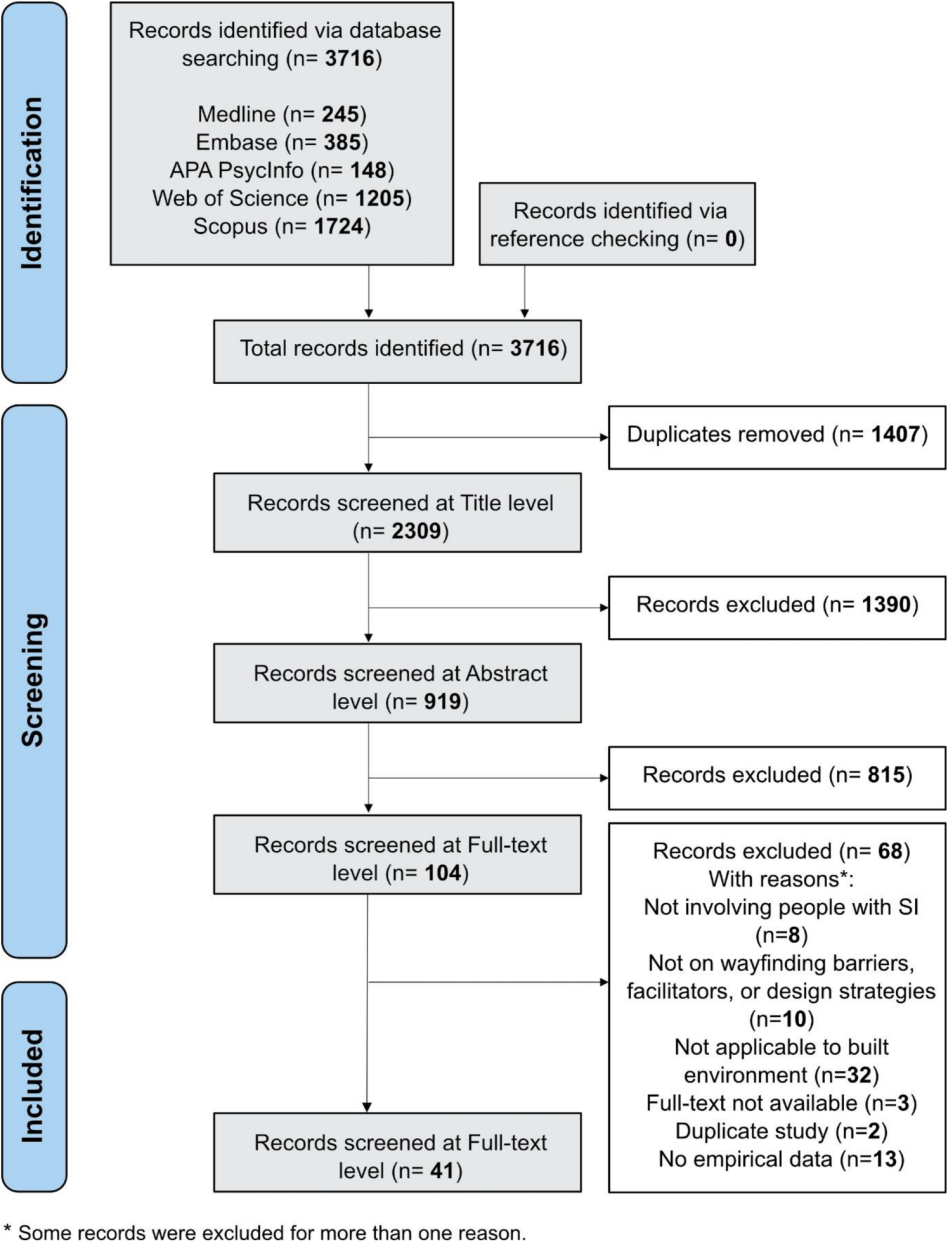
Flow Diagram of Scoping Review Data According to the Preferred Reporting Items for Systematic Reviews and Meta-Analyses Extension for Scoping Reviews (PRISMA-ScR).

In total, 3,716 records were identified, and after duplicate removal, 2,309 record titles, 919 abstracts, and 104 full texts were screened. In total, 41 studies were included in the review (Figure 2).

### Description of Studies

**Figure 3.**
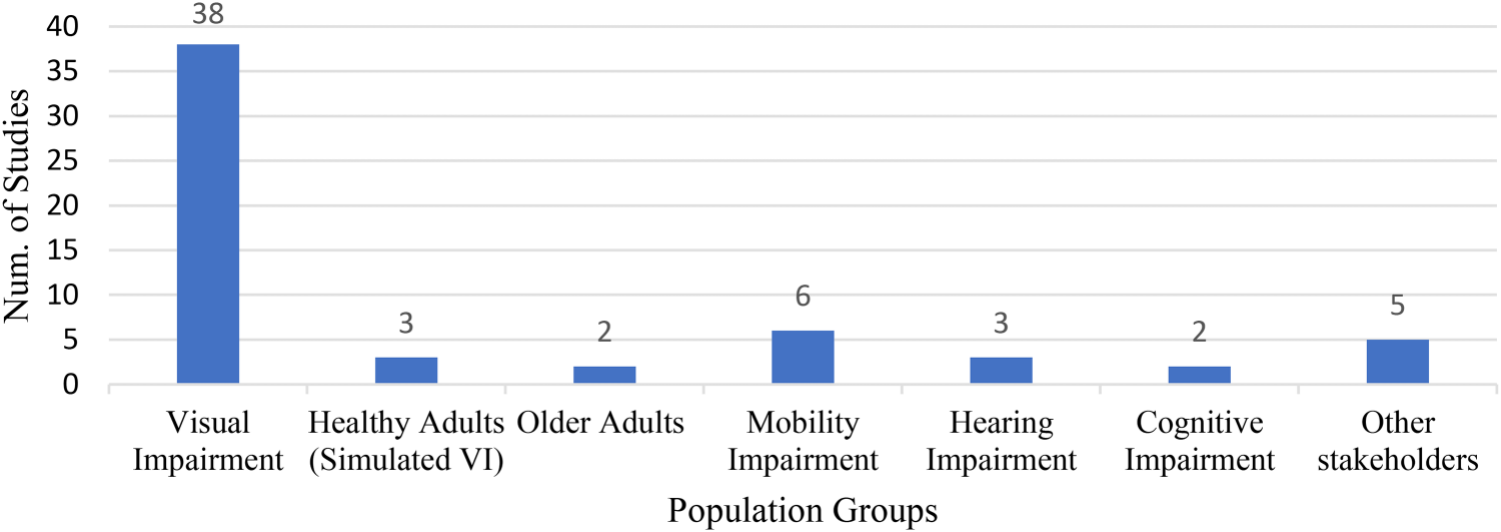
Distribution of Population Groups Among Included Studies (*n* = 41).

The studies were conducted in 19 countries: USA (7), United Kingdom (5), Turkey (4), Greece (4), Italy (3), Australia (3), Japan (2), Germany (2), and Malaysia, South Korea, Slovakia, Taiwan, France, Thailand, Israel, Prague, Russia, Mexico, and Portugal accounted for one study each. All the included studies involved people with visual impairments, including three that involved healthy adults with simulated visual impairment (Belir, 2018; Belir, 2021; Rousek & Hallbeck, 2011b). While most studies (*n* = 29) focused solely on visual impairment, the remainder (*n* = 12) involved multiple impairments (hearing/ mobility/ cognitive impairments, older adults) and other stakeholders (such as built environment experts, ophthalmic professionals, and orientation and mobility officers) (Figure 3). Participants with hearing impairment were involved in three studies with multiple user groups (Gupta et al., 2020; Han et al., 2020; Morag et al., 2016), but none of the studies focused on their orientation needs and challenges.

The context of studies included diverse settings within the built environment. Many studies (*n* = 16) examined urban spaces, and 13 explored built environments in general without specifying indoors or outdoors. The 12 remaining studies focused on buildings and indoor environments. Among these, four studies explored healthcare settings, and the remainder explored public buildings (*n* = 4), transport buildings (*n* = 1), educational settings (*n* = 1), and music venues (*n* = 1). For the purpose of this article, the 25 studies exploring built environments or indoors are discussed (Table 1). A summary of the remaining 16 studies on urban spaces is presented in the Supplementary Materials (Appendix II).

**Table 1.** Characteristics of Included Studies on the Built Environment and Indoors (*N* = 25) (Ordered Chronologically).

Author (Year), Data Location	Population	Concept (Aim)	Context	Study Design, Methods
Nagassa et al. (2023), Australia	VI *N* = 9	Evaluating the usefulness of 3D models of buildings in cognitive mapping for people with VI	Public buildings	Mixed methods, usability test, questionnaire, observation, and open-ended interview
Castle et al. (2022), United Kingdom	VI adults *N* = 20 (S1) *N* = 94 (S2)	Accessibility requirements of music events for people with VI	Music venues	Study 1: structured interview Study2: online survey
Müller et al. (2022), Germany	People with low vision, blindness, or mobility impairments *N* = 125	To examine the travel behaviour for both trip planning and execution, for people with impairments	Indoor environment	Quantitative, survey
Manandhar et al. (2022), Australia	VI, *N* = 28 (15 normal vision and 13 VI)	To examine the preferred luminance contrast of four building elements for people with VI	Built environment	Quantitative, experiment
Belir (2021), Turkey	Simulated VI *N* = 27 (blindfolded, and observer)	Understanding the impact of sensory landmarks on spatial legibility	Indoor environment	Mixed methods, navigation task and space syntax analysis
Im and Sang (2021), Malaysia	VI (students aged 13–26) Sample size not mentioned	Understanding sensory perceptions of the visually impaired and their challenges in cognitive learning	Built environment (focused on school spaces)	Qualitative, in-depth interviews and observation
Swaminathan et al. (2021), United States	VI (*N* = 67), accessibility experts (*N* = 10)	Investigating the role of tactile cues in nonvisual navigation and augmenting existing tactile surfaces with multimodal feedback	Built environment	Mixed methods, survey of people with VI, interview with experts
Engel (2020), Germany	VI *N* = 106	Wayfinding strategies among people with low vision (LV) and blindness (BL)	Indoor environment (unfamiliar buildings)	Quantitative, survey
Gupta et al. (2020), United States	*N* = 27 Older adults, VI, HI, MI, CI	Examining urban navigational experiences and preferences of people with impairments	Built environment	Qualitative, interviews
Han et al. (2020), South Korea	People with MI, VI, HI *N* = 18	To design a smart accessibility data model that integrates geospatial data with environmental accessibility	Built environment	Qualitative, direct observation and activity mapping, data modeling
Toyoda et al. (2020), Japan	VI *N* = 20	To clarify the impact of combining verbal explanation with 3D tactile maps on independent navigation	Built environment (unfamiliar)	Mixed methods, navigation task, interviews
Jeamwatthanachai et al. (2019b), Thailand	VI (*N* = 30) and experts (*N* = 15)	To explore VI individuals’ behaviors and strategies for navigating indoor public spaces	Indoor environment (public buildings)	Mixed methods, questionnaire and semistructured interviews
Jeamwatthanachai (2019a), United Kingdom	Experts *N* = 15	To develop a building rating system (BRS) to classify the accessibility of buildings for VI	Indoor environment	Mixed methods, expert validation and user evaluation (site inspection and focus groups)
Afshary et al. (2019), Italy	VI aged 30–60 *N* = 8	To explore how focus groups enhance vocabulary on VI capacities and environmental legibility/complexity	Built environment	Qualitative, focus group
Belir (2018), Turkey	Simulated VI *N* = 6 (blindfolded/observer)	The importance of using nonvisual senses and their role in architectural education	Built environment	Qualitative, field experiment and blindfolded navigation
Zhao et al. (2018), United States	VI *N* = 14	Understanding the perceptions of people with VI when navigating surface-level changes	Built environment	Qualitative, structured interviews, observation of participants, and think-aloud method
Morag et al. (2016), Israel	People with diverse needs (VI, HI, MI, CI, nonlocal language) *N* = 56	To develop and assess a questionnaire for identifying wayfinding challenges among diverse hospital users	Healthcare (hospitals)	Mixed methods, evaluation methodology using a questionnaire
Fixova (2014), Czech Republic	*N* = 12 VI and older people (over 60)	To analyze the needs of people with limited orientation and develop an adaptive navigation system	Healthcare (public hospitals)	Qualitative, field study and usability tests
Korček and Rollova (2014), Slovakia	VI	Investigating the current condition of the Slovak transport system regarding wayfinding for VI	Transport buildings	Qualitative, case study (access audit of selected transport buildings)
McIntyre and Hanson (2014), United Kingdom	VI *N* = 10	Presenting a framework for the analysis of the interaction between the built environment and VI users	Public buildings	Mixed methods, semistructured interviews, wayfinding tasks, and observation
Huang and Yu (2013), Taiwan	VI *N* = 7 adults aged 20–22, all white cane users	Evaluation of architectural elements and designs helping the blind or visually impaired in wayfinding by studying their environmental perception patterns	Built environment	Mixed methods, Q methodology (a method used to collect subjective opinions and conduct quantitative analysis)
Belir and Onder (2013), Turkey	VI *N* = 14	To investigate the impact of spatial design (location of landmarks) on the cognitive mapping ability of people with VI	Public buildings (shopping malls)	Mixed methods, Navigation task and direct observation, Space syntax analysis
Rousek and Hallbeck (2011b), United States	Simulated VI (healthy adults wearing simulation goggles) *N* = 50, aged 18–30	Analyzing the current issues in a wayfinding task for the VI and normally sighted to identify wayfinding design deficits	Healthcare (hospitals)	Mixed methods, navigation task followed by questionnaire, statistical analysis
Rousek and Hallbeck (2011a), United States	VI, aged 18–30 *N* = 50	To assess the effectiveness of current healthcare signage, particularly focusing on pictogram designs, especially for people with VI	Healthcare	Quantitative, questionnaire, including tasks for identifying and rating pictograms, and statistical analysis
Thapar et al. (2004), United States	*N* = 4 VI, MI, control with no impairment	Comparing functional access to public buildings and facilities for people with and without impairments	Public buildings	Mixed methods, navigation task, survey

VI = visual impairment; HI = hearing impairment; MI = mobility impairment; CI = cognitive impairment.

### Critical Appraisal

Studies were assessed based on the quality criteria specific to their methodology in MMAT by one researcher (PZ). Overall quality scores were calculated based on the percentage of corresponding criteria met (Hong, Fàbregues et al., 2018), and the results are presented in the Supplemental Materials (Appendix III). Only five studies met all quality criteria for their respective designs, one of which had a quantitative design (Manandhar et al., 2022) and the other four used a qualitative design (Cushley et al., 2022; Han et al., 2020; Rey-Galindo et al., 2020; Zhao et al., 2018). The quality of 10 studies (see Supplementary Materials, Appendix III) could not be assessed, mainly due to the lack of clear research questions.

### Summary of Findings

The review highlights the key environmental factors impacting wayfinding and navigation among individuals with SI, perceived barriers and facilitators, and design strategies to enhance access for this population. The findings related to urban accessibility are not reported in this article. According to the conceptual framework (Figure 1), the results are presented in three main categories: architectural elements, information/ graphical elements, and sensory elements (see Table 2).

**Table 2.** Environmental Features That Impact Wayfinding Experiences of People With Sensory Impairments and User Perceptions; (B) Barriers, (F) Facilitators.

Categories	Subcategories	Structural Element	User Perceptions (Barriers/Facilitators)	Sources
Architectural elements	Regions/plan configuration	Symmetrical layout	(B) Being disoriented due to uniform and symmetrical layout	(Belir, 2021; Belir & Onder, 2013)
		Grid vs. circular layout	(B) Nonright-angled corners and curved hallways causing disorientation (F) Preference for grid layouts for lower complexity	(Gupta et al., 2020; Rousek & Hallbeck, 2011b)
		Wide-open spaces	(B) Challenging for wayfinding due to the lack of sensory (visual and nonvisual) landmarks or information	(Jeamwatthanachai et al., 2019b; Rousek & Hallbeck, 2011b)
		Intermediate or half-floors	(B) Causing disorientation for people with VI	(Müller et al., 2022)
	Edges/entrances and exits	Outdoor access to the building	(B) Difficulty in identifying the entrance due to the lack of a defined pathway from the parking or the drop-off point	(Castle et al., 2022; McIntyre & Hanson, 2014)
		Visibility of entrances/exits	(B) Glazed facades/doors without clear markings making the entrance less visible	(Korček & Rollova, 2014)
		Automatic vs. revolving doors	(B) Revolving doors and malfunctioning automatic doors perceived as hazardous (F) Preference for automatic doors over other types	(Han et al., 2020; Jeamwatthanachai et al., 2019b; McIntyre & Hanson, 2014)
	Paths and horizontal circulation		(B) Feeling confused due to the complexity of the layout and lack of clear circulation; complicated paths when navigating from one destination to another in a hospital (F) Corridors helping some people feel safe and concentrate on their route	(Afshary et al., 2019; McIntyre & Hanson, 2014; Morag et al., 2016; Thapar et al., 2004)
	Vertical access (stairs/lifts/escalators)	Visibility and proximity	(B) Low visibility of stairs, especially on a glass staircase (F) Being notified of the location of vertical access upon entering a building	(Gupta et al., 2020; Müller et al., 2022)
		Usability	(B) The small number of working lifts; being afraid of using stairs (F) Preference for using ramps	(Gupta et al., 2020)
		Adherence to standards and design	(B) Inconsistent dimensions of stairs create uncertainty and safety concerns, missing marking of the first and last steps, curved design, and lack of color contrast between the riser and tread, reducing visibility	(Jeamwatthanachai et al., 2019b; Korček & Rollova, 2014; Zhao et al., 2018)
		Light and shadows	(B) Insufficient lighting in the staircase makes it hard to detect stair edges; shadows are perceived as steps; transparent glass walls creating disorientation due to reflections	(Zhao et al., 2018)
		Sensory features	(F) Availability of sensory features such as braille on the handrails, help buttons, and voice output in lifts, stairs, and escalators; bend at the end of the handrail indicating the stairs’ start/end and direction	(Han et al., 2020; Müller et al., 2022)
	Nodes and intersections (decision points)	Visibility of nodes	(B) Identifying key facilities such as service desks, toilets and bars	(Castle et al., 2022)
	Landmarks and furniture	Availability and location	(B) Lack of or inconsistent placement of landmarks (F) Familiar and predictable landmarks; accurately and strategically located landmarks improving the legibility of space for VI	(Belir, 2021; Belir & Onder, 2013)
		Sensory landmarks	(F) landmarks that engage with multiple senses	(Belir & Onder, 2013)
		Presence of obstacles	(B) Presence of obstacles in the pathway, especially those at body or head level (e.g., overhanging signs), which are difficult to detect with a cane, but also moving obstacles like trolleys; Furniture arranged in “island” layout	(Jeamwatthanachai et al., 2019a; Müller et al., 2022; Rousek & Hallbeck, 2011b)
Information/graphical elements	Signage	Availability	(B) lack of signage and directions in public buildings; overabundance of visual signs that create a visual overload	(Afshary et al., 2019; Thapar et al., 2004)
		Adherence to standards and design	(B) Improper illumination of the sign, unexpected positioning, signs being small, small lettering, and low tonal contrast (F) Use of pictograms on signs; pictograms with greater contrast, less complexity, and fewer abstract features	(Han et al., 2020; Müller et al., 2022; Rousek & Hallbeck, 2011b, 2011a)
	Maps	Tactile maps	(F) 3D tactile maps as a useful tool for creating a cognitive map of a building before visiting it	(Nagassa et al., 2023; Toyoda et al., 2020)
Sensory elements	Lighting	Intensity	(B) Insufficient lighting reducing the visibility, making it difficult to identify obstacles, find destinations, and read signs; difficulty in lip-reading for people with HI due to low lighting levels	(Morag et al., 2016; Rousek & Hallbeck, 2011b; Thapar et al., 2004; Zhao et al., 2018)
		Consistency	(B) Sharp contrast from light to dark when entering venues; inconsistency in lighting along the route, and sharp natural light from windows causing glare (F) Strobe lighting for people with HI	(Castle et al., 2022; Han et al., 2020)
	Visual contrast	Contrast levels	(B) lack of or low color and tonal contrast between surfaces; unexpected changes in color or changes for aesthetic purposes could be misinterpreted as changes in depth; using colors difficult to distinguish for those with color blindness	(Afshary et al., 2019; Müller et al., 2022; Zhao et al., 2018)
	Soundscape (auditory cues)	Availability	(F) Presence of audio feedback on lifts and other facilities; acoustic navigation systems; using sound to create a sense of depth, distance, and movement	(Afshary et al., 2019; Huang & Yu, 2013)
		Intensity	(B) Excessive noise in crowded areas creating distraction and loss of orientation for VI and HI; overly quiet environments creating anxiety as individuals receive less auditory feedback	(Jeamwatthanachai et al., 2019b)
	Textures (Tactile cues)	Contrast	(B) Doors with the same texture as the surrounding wall, undifferentiated texture in open areas; lack of contrast between floor and wall	(Afshary et al., 2019; Korček & Rollova, 2014)
		Materials	(B) Carpet deafens the echo of white canes; glass walls and glass stairs, and shiny flooring causing disorientation/glare	(Gupta et al., 2020; McIntyre & Hanson, 2014; Zhao et al., 2018)
	Olfactory cues		(F) Using different smells to navigate in a building (e.g., smell of coffee)	(McIntyre & Hanson, 2014)

#### Architectural Elements

The plan configuration and layout of the building are discussed in several studies as having a significant impact on wayfinding, particularly for people with VI. Symmetrical layouts were found to be confusing because their uniformity made it difficult to distinguish one area from another for individuals with sight loss (Belir, 2021; Belir & Onder, 2013). Similarly, circular layouts and those with curved hallways posed challenges for users to maintain their orientation and navigate effectively (Rousek & Hallbeck, 2011b). Several participants in a study, including people with VI and older adults, preferred grid layouts over curved ones as grid layouts were easier to understand and more intuitive to navigate for them (Gupta et al., 2020). Wide-open spaces such as lobbies and open plazas were problematic, as they lacked sufficient landmarks or sensory cues that are essential for orientation with SI (Jeamwatthanachai et al., 2019b; Rousek & Hallbeck, 2011b). Additionally, features like intermediate or half-floors were perceived as highly challenging for users with VI, often leading to confusion and increased disorientation (Müller et al., 2022).

The approach to the building, entrances, and exit doors is another important factor involved in wayfinding. A common barrier found was the lack of a disabled parking area and a clearly defined route from the parking area or drop-off point to the entrance, which can make locating the entry point challenging (Castle et al., 2022; McIntyre & Hanson, 2014). The entrance or exit doors should be clearly marked. Korček and Rollova (2014), found that the use of glazed facades or doors without adequate markings poses barriers for people with VI and suggested using a colored line to frame the glazed door or eye-level graphical markings to improve their visibility. The type of door mechanism also affected accessibility; revolving doors and malfunctioning automatic doors were perceived as unsafe or difficult to use. Automatic doors were preferred and generally seen as more accessible for users with VI (Han et al., 2020; Jeamwatthanachai et al., 2019b; McIntyre & Hanson, 2014).

Several studies addressed the accessibility of paths and the horizontal circulation of the building. Locating destinations in a building without a clear circulation was confusing for several participants (Afshary et al., 2019; Thapar et al., 2004). However, corridors helped a few users with VI to feel safe and concentrate on their wayfinding (McIntyre & Hanson, 2014). Within a hospital setting, Morag et al. (2016) found that locating multiple destinations was complicated and time-consuming for participants (including users with VI and HI), and they were unable to follow the colored arrows.

The vertical access points, including stairs, lifts, and escalators, were frequently discussed in the literature, presenting both barriers and facilitators for individuals with VI. A significant barrier was the low visibility of the stairs, particularly glass staircases, where the stair edges can be difficult to detect. This can be compounded by insufficient lighting and misleading shadows that may be perceived as steps (Müller et al., 2022; Zhao et al., 2018). The inconsistent dimensions of stairs, lack of color contrast between risers and treads, and absence of markings on the first and last steps further contribute to uncertainty and safety concerns (Jeamwatthanachai et al., 2019b; Korček & Rollova, 2014). Functional issues, such as a limited number of operational lifts and anxiety related to stair use, also hindered accessibility and resulted in a preference for ramps (Gupta et al., 2020). However, certain design features acted as facilitators. This included high-contrast stripes on step nosing, consistent lighting to improve the visibility of stairs, and avoiding curved stairs (Zhao et al., 2018). Braille signs on handrails could communicate the floor numbers, and the bend at the end of the handrail signified the end of the staircase. Furthermore, the availability of help buttons and voice output systems in lifts increased the accessibility and safety for VI users (Han et al., 2020; Müller et al., 2022).

Finally, decision points (nodes) and landmarks were other key architectural features in wayfinding with SI. In a study by Castle et al. (2022), visually impaired users reported problems with locating key facilities (e.g., service desks, toilets, and bars) in public music venues. While inconsistent or insufficient placement of landmarks constituted a barrier, the presence of predictable and strategically located landmarks enhanced the spatial legibility, especially those that engaged with multiple senses. Additionally, the placement of structural and sensory landmarks along main pathways helped improve cognitive mapping for visually impaired users (Belir, 2021; Belir & Onder, 2013). The presence of obstacles within the pathway further compounded the challenges of navigation for this population. Barriers included static obstacles (such as pillars or information boards), objects at body or head level (such as overhanging signs or furniture), which are often undetectable by a white cane, as well as moving objects like trolleys (Jeamwatthanachai et al., 2019a; Müller et al., 2022; Rousek & Hallbeck, 2011b).

#### Information and Graphical Elements

The literature highlighted several considerations regarding the signage. A recurring barrier identified was the inconsistency in signage availability, with some public buildings lacking adequate directions while others present an overwhelming abundance of signs, leading to visual overload (Afshary et al., 2019; Thapar et al., 2004). In terms of signage design, barriers included improper illumination, unexpected placement, limited tonal contrast, high placement, and small lettering, all of which reduce the legibility of the signs (Müller et al., 2022; Rousek & Hallbeck, 2011b). Han et al. (2020) found that the use of pictograms on signs is beneficial for Deaf individuals who rely on sign language services and might have low literacy levels. Rousek and Hallbeck (2011a) recommended consistency and standardization in signage design across healthcare facilities, emphasizing the use of human-based figures to enhance comprehension, minimize complexity, and maximize color contrast.

Tactile maps and 3D models of buildings were found to be effective for enhancing the cognitive mapping of users with VI. Incorporated into orientation and mobility (O&M) training, they allowed users to build a mental representation of an environment before physically visiting it (Nagassa et al., 2023; Toyoda et al., 2020).

#### Sensory Elements

The sensory features of the environment, including the visual, auditory, tactile, and olfactory cues, have a profound effect on the wayfinding experience of individuals with SI.

In the visual domain, lighting was discussed as a primary concern. Studies found that low lighting levels reduced the visibility of obstacles and signage and impeded communication for individuals with HI who relied on lip-reading (Morag et al., 2016; Rousek & Hallbeck, 2011b; Thapar et al., 2004; Zhao et al., 2018). Additionally, inconsistencies in lighting, such as sudden transitions from bright to dark areas or glare from windows, can disorient users and disrupt the continuity of their navigation. Therefore, evenly distributed lighting is a key factor in wayfinding, especially for users with impaired vision. Also, targeted lighting interventions, such as strobe lighting, might help people with HI by providing nonverbal directional signals (Castle et al., 2022; Han et al., 2020).

Complementing these findings, visual contrast was determined to be crucial, particularly for people with some residual vision. Low color and tonal contrast reduced the visibility of surfaces for users with VI. Studies suggested that contrast should be used intentionally; sudden or purely decorative changes on floors could create confusion and be misinterpreted as level changes and increase the risk of falls and trips, especially in older adults (Afshary et al., 2019; Müller et al., 2022; Zhao et al., 2018). Manandhar et al. (2022) evaluated the efficiency of the current Australian requirement of 30% luminance contrast for building elements (Australian Standard, 2021), equivalent to the 30-point Light Reflectance Value (LRV) contrast requirement in the UK (BS 8300-2:2018). They emphasized the need to increase the luminance contrast levels in building elements such as door frames and light switches, going beyond the current 30% requirement to around 65% for better visibility for those with severe VI.

The auditory environment or soundscape further contributes to the navigational experience. Facilitators included the presence of auditory cues, such as audio feedback in lifts, acoustic navigation systems, and ambient sounds that help users perceive spatial depth and movement (Afshary et al., 2019; Huang & Yu, 2013). While overly quiet environments may fail to provide necessary auditory feedback, excessive ambient noise in crowded areas can be overwhelming and disorienting for people with SI (Jeamwatthanachai et al., 2019b).

Tactile elements are equally essential in wayfinding, especially for users with severe sight loss. Changes in materials, textures, and terrain slopes provide tactile cues for wayfinding (Huang & Yu, 2013), and tactile paving could be used to guide users towards key features like staircases (Korček & Rollova, 2014). A lack of tactile contrast—such as doors blending into walls or floors that do not differ in texture from surrounding areas—impairs spatial perception (Korček & Rollova, 2014). Improper selection of surface materials poses barriers; for example, carpets can dampen the auditory feedback from mobility aids, and shiny surfaces can cause visual disorientation or glare (Gupta et al., 2020; McIntyre & Hanson, 2014; Zhao et al., 2018).

Lastly, olfactory cues, though less commonly discussed in literature, show promise of facilitating wayfinding. Familiar and distinct smells (e.g., the scent of coffee near a café) can serve as effective spatial markers for people with VI (McIntyre & Hanson, 2014).

## Discussion

The results of this review illustrate the multidimensional nature of wayfinding design and highlight the specific access needs of people with visual and/or hearing impairments. These can provide a robust evidence base for updating the key accessibility standards, such as the British Standards (BS 8300-2:2018) and the Americans with Disabilities Act (ADA) Standards (Department of Justice, 2010). The findings reinforce the principles within current accessibility guidelines (British Standards Institution, 2021), underscoring the necessity of integrating wayfinding design throughout the entire building lifecycle. This holistic approach demands that wayfinding be a key consideration from the earliest stages of conceptual design and business case development, through construction and post-occupancy assessments.

*The results of this review illustrate the multidimensional nature of wayfinding design and highlight the specific access needs of people with visual and/or hearing impairments*.

The conceptual framework emerging from this review encourages stakeholders to shift their perception of wayfinding design from solely reliance on signage to a holistic approach that fully integrates the architectural and spatial, graphical, and sensory characteristics of the built environment.

Of particular importance is the impact of spatial design and layout on user orientation, a factor that remains under-addressed in many accessibility guidelines. The evidence indicates a strong preference among people with VI for predictable, grid-based layouts over curved designs. To mitigate the disorientation caused by uniform and symmetrical spaces, designers should incorporate distinct identifying features, such as high-contrast colors, clear graphics, or tactile markers. Similarly, wide-open spaces require the integration of sensory landmarks to provide crucial orientation cues. Building on these findings, further research is warranted to investigate how various architectural layouts impact the wayfinding behaviors of people with SI.

*Of particular importance is the impact of spatial design and layout on user orientation, a factor that remains under-addressed in many accessibility guidelines*.

Regarding signage design, while the high placement and small lettering were recognized as barriers, no empirical evidence was provided to determine the optimal lettering size and location of signs for people with reduced vision. The ADA guidelines (U.S. Access Board, 2010) mandate a mounting height of 48–60 inches (approximately 122–152 cm) for tactile signs, and specify that character height for visual signs should be based on viewing distance to ensure legibility. Nevertheless, these standards may not sufficiently accommodate the needs of older adults, who represent a majority of visually impaired users. Given the natural tendency for a lower eye level and downward gaze in older people, the efficacy of the current standards is questionable. Therefore, empirical research is needed to validate or refine these guidelines, focusing specifically on accessible signage for this population.

Regarding sensory features, although visual contrast is found to be essential in facilitating navigation, its effectiveness is affected by the level of contrast and quality of light (British Standards Institution, 2021). Reliance on color contrast alone is not sufficient, as it excludes individuals with color vision deficiency or severe VI. Therefore, tonal contrast—often defined by the LRV of the surfaces—should be prioritized and complemented with tactile elements such as braille and embossed features.

## Directions for Future Research

This scoping review highlights significant gaps regarding the participant groups studied. Notably, the specific wayfinding needs and experiences of individuals with HI remain largely underexplored, with only three studies involving this group and none focusing on them exclusively. Future research can investigate the experiences and wayfinding challenges of people with HI and design factors supporting their orientation and mobility. Furthermore, the exclusion or underrepresentation of older adults, mainly due to recruitment difficulties, presents a critical knowledge gap. Given that sensory deterioration is often an inevitable part of the aging process, research targeting older adults is essential to understand their unique accessibility requirements and ensure wayfinding strategies cater to this growing demographic.

*Given that sensory deterioration is often an inevitable part of the aging process, research targeting older adults is essential to understand their unique accessibility requirements.*

The existing body of literature mainly focuses on identifying barriers encountered by individuals with SI during wayfinding. While understanding these is crucial, there is a pressing need for future research to shift toward exploring and validating design solutions. Experimental studies are required to examine the efficacy of specific design elements (e.g., circulation systems, placement of landmarks, levels of lighting) on the wayfinding performance of people with SI, which can lead to developing evidence-based guidelines and interventions.

Finally, this review identified limited evidence regarding the role of tactile and olfactory cues in wayfinding for individuals with SI, despite providing crucial information for navigation. Future research should delve into how people with SI use tactile and olfactory cues to develop a mental map of the building.

## Strengths and Limitations

This study is, to our knowledge, the first scoping review presenting the wayfinding needs of people with SI. The developed conceptual framework, grounded in established theories (Arthur & Passini, 1992; Lynch, 1960) and enriched through content analysis, integrates environmental features with subjective user perceptions, offering a holistic perspective to enhance built environment accessibility. The research strategy was not limited to any specific languages or locations, so it provides a comprehensive global overview of the available scholarly knowledge on this topic. Despite these strengths, the review is subject to certain limitations. While the search strategy did not include gray literature, the results are discussed in relation to the current guidelines. Critical appraisal, although not an essential component of this scoping review, was performed by one researcher. Finally, as with any review process involving qualitative synthesis, an element of researcher interpretation is unavoidable, although efforts were made to ground this systematically in the review process.

## Conclusion

This scoping review systematically mapped the scholarly evidence on the environmental factors that influence wayfinding for individuals with visual and/or hearing impairments. It also identified key design recommendations to improve built environment accessibility for this population. A central contribution is the conceptual framework presented, which organizes key environmental features that impact wayfinding for people with SI. The framework invites stakeholders, including interior designers, architects, and policymakers, to consider wayfinding design from a holistic perspective as an interplay between architectural and graphical components and the sensory needs of users, moving beyond relying solely on signage. These findings highlight the importance of various environmental features in wayfinding design and inform the development of accessibility guidelines for people with sensory loss alongside all users.

*The framework invites stakeholders, including interior designers, architects, and policymakers, to consider wayfinding design from a holistic perspective as an interplay between architectural and graphical components and the sensory needs of users, moving beyond relying solely on signage*.

## Implications for Practice

- Wayfinding design requires a holistic approach that integrates the architectural, graphical, and sensory characteristics of the built environment.
- Empirical evidence indicates a strong preference among the visually impaired for grid-based layout over curved designs.
- Given the tendency of a downward gaze in older adults, current standards for mounting heights of signage may not adequately accommodate the needs of this population.
- Reliance on color contrast alone is insufficient and designers should prioritize tonal contrast.

## Supplemental Material

sj-docx-1-her-10.1177_19375867251391361 - Supplemental material for A Scoping Review of the Impact of Environmental Design on Wayfinding for People With Sensory Impairment Supplemental material, sj-docx-1-her-10.1177_19375867251391361 for A Scoping Review of the Impact of Environmental Design on Wayfinding for People With Sensory Impairment by Parastoo Zali, Lori B. McElroy, Mario Ettore Giardini, Kullapat Chaiyawat and Margaret Watson in HERD: Health Environments Research & Design Journal
