# Supplementary material for: A Scoping Review of the Impact of Environmental Design on Wayfinding for People With Sensory Impairment
Source: HERD. 2025 Nov 20;19(2):48–67. doi: 10.1177/19375867251391361 (PMC12988013; doi:10.1177/19375867251391361)
Supplement: sj-docx-1-her-10.1177_19375867251391361 - Supplemental material for A Scoping Review of the Impact of Environmental Design on Wayfinding for People With Sensory Impairment [file sj-docx-1-her-10.1177_19375867251391361.docx]

**Supplementary Materials**

Appendix I: Search strategy used for Medline (Ovid)

| # | Topic | Query | Num. of Results |
| --- | --- | --- | --- |
| 1 | Sensory impairment | (sensory impair* or visual impairment or visually impaired or hearing impairment or hearing impaired or dual impair* or dual sensory impair* or sight impair* or visual disorder or vision disorder or hearing disorder or deaf* or blind* or deaf blind* or vis* loss or sight loss or hearing loss or proprioception impair*).mp. | 567639 |
| 2 | (MeSH term) | exp Vision Disorders/ | 79112 |
| 3 |  | exp Hearing Loss/ | 77821 |
| 4 | All sensory impairments | 1 or 2 or 3 | 611272 |
| 5 | Task | (navigation* or wayfinding or way finding or pathfinding or spatial orientation or spatial perception).mp. | 42551 |
| 6 | (MeSH term) | exp Space Perception/ or exp Orientation, Spatial/ | 86524 |
| 7 | All Tasks | 5 or 6 | 125746 |
| 8 | Context | (healthcare or hospital* or care home* or nursing home* or pharmacy or domestic or housing or residential or urban or built environment or (inclusive adj3 environment) or (accessible adj3 environment) or architectur* or indoor* or outdoor*).mp. | 2713831 |
| 9 | (MeSH term) | exp Environment Design/ or exp Built Environment/ | 8448 |
| 10 | All Contexts | 8 or 9 | 2717406 |
| 11 | All Result | 4 and 7 and 10 | 344 |
| 12 | Time limit | limit 11 to yr="2000 -Current" | 312 |
| 13 |  | limit 12 to humans | 254 |

Appendix II – Characteristics of the included studies on urban spaces (N=16) (ordered chronologically); VI = Visual Impairment

| Author (Year),  Data Location | Population | Concept (Aim) | Study Design, Methods |
| --- | --- | --- | --- |
| Cushley et al., (2023)  United Kingdom | VI and built environment professionals N=108 (survey)  N= 8 (interviews) | To identify challenges for visually impaired people and how professionals address them | Mixed methods, Questionnaires (main method), focus groups, and interviews |
| Cushley et al., (2022)  United Kingdom | VI, N=20  built environment and ophthalmic professionals,  and sight loss charities | Identifying the barriers and enablers to navigating streets with a visual impairment | Qualitative,  Semi-structured interviews |
| Campisi et al., (2021)  Italy | VI Adult (>18) N=79 (First phase) N=21 (Second phase) | assessment of the walkability of urban spaces from the perspective of visually impaired people | Mixed methods, Phase1: Questionnaire  Phase2: Commented Paths method (CPM) including subjective evaluation  Phase3: Calculation of walkability indices |
| Синицын & Запорожец, (2021)  Russia | Blind people  N=10 | Analysis of the orientation and navigation techniques employed, and barriers perceived by blind individuals in urban settings | Qualitative,  Semi-structured interviews |
| Kan‐Kilic et al., (2020)  Portugal | Congenitally blind people  N=5 | Wayfinding strategies of blind people in the urban environment; Assessing the importance of auditory cues in wayfinding | Mixed methods,  wayfinding task and concurrent verbalization |
| Lukman et al., (2020)  Australia | VI  N=12 | Examining whether the level of luminance contrast in Australian accessibility standards (30%) is adequate for people with VI to detect and identify tactile indicators | Quantitative,  lab-based experiment with users |
| Rey-Galindo et al., (2020)  Mexico | VI  N=18  Adults, aged 21-80 | To identify the information needs of people with VI while performing daily activities using public space | Qualitative,  Semi-structured interviews |
| Papadopoulos et al., (2018)  Greece | Blind people  N=21 | Examining the ability  of blind individuals to create cognitive maps of routes in an unfamiliar area through the use of tactile and audio-tactile maps. | Quantitative,  Questionnaire and navigation task |
| Kan‐Kilic & Dogan, (2017)  Turkey | Congenitally blind people  N=9 | Examining the impact of urban environment’s sensory cues on the wayfinding strategies of blind people | Mixed methods,  wayfinding task and concurrent verbalization |
| Secchi et al., (2017)  Italy | Blind people  N=24 | Measuring and analyzing acoustic contrast between different paving materials as a cue for the wayfinding of blind people | Mixed methods,  Field experiments with participants, on-field measurements |
| Omori et al., (2015)  Japan | VI  N=15 | Evaluating the effectiveness of two types of voice guide and graphic floor signs | Mixed methods,  Lab-based experiment with users, direct observation |
| Tajgardoon & Karimi, (2015)  United States | VI and wheelchair users | A new approach to evaluating the accessibility of built environments (sidewalk networks) for wayfinding  of individuals with disabilities | Quantitative,  Simulation, modeling, and visualization |
| Koutsoklenis & Papadopoulos, (2014)  Greece | VI  1. N=9, Aged 20-38  2. N=32, Aged 18-56  3. N=15, Aged 20-56 | Investigating the most significant haptic cues used for urban wayfinding for people with VI and how they use these cues | Mixed methods,  1. Focus-groups interview  2. Questionnaires  3. Structured interview |
| Koutsoklenis & Papadopoulos, (2011)  Greece | VI  1. N=9, aged 20-38  2. N=32, aged 18-56  3. N=15, aged 20-56 | Investigating the most significant olfactory cues used for urban wayfinding for people with VI and how they use these cues | Mixed methods,  1. Focus-groups interview  2. Questionnaires  3. Structured interview |
| Koutsoklenis & Papadopoulos, (2011a)  Greece | VI  1. N=10, Aged 20-38  2. N=60, Aged 17-79  3. N=20, Aged 20-56 | Investigating the most significant auditory cues used for urban wayfinding for people with VI and how they use these cues | Mixed methods,  1. Focus-groups interview  2. Questionnaires  3. Structured interview |
| Picard & Pry, (2009)  France | VI  N=10 | To assess the efficiency of a model of a familiar urban area for enhancing knowledge of the spatial environment by adults with VI | Quantitative,  Spatial tasks and model exposure, with statistical analysis |

Appendix III – Critical appraisal of included studies using Mixed Methods Appraisal Tool (ordered by quality scores)

(Y= Yes, N=No, C= Can’t tell, N/A = Not Applicable)

| **Author, Year** | **Screening Questions** | | **Qual. component** | | | | | **Quan. component** | | | | | **Mixed methods component** | | | | | **Comments** | **Score**  **(%)** |
| --- | --- | --- | --- | --- | --- | --- | --- | --- | --- | --- | --- | --- | --- | --- | --- | --- | --- | --- | --- |
|  | S1 | S2 | 1.1 | 1.2 | 1.3 | 1.4 | 1.5 | 4.1 | 4.2 | 4.3 | 4.4 | 4.5 | 5.1 | 5.2 | 5.3 | 5.4 | 5.5 |  |  |
| Cushley et al., (2022) | Y | Y | Y | Y | Y | Y | Y |  |  |  |  |  |  |  |  |  |  |  | 100 |
| Manandhar et al., (2022) | Y | Y |  |  |  |  |  | Y | Y | Y | Y | Y |  |  |  |  |  | Power calculation was performed to ensure the adequacy of the sample size. Various types of VI included. | 100 |
| Han et al., (2020) | Y | Y | Y | Y | Y | Y | Y |  |  |  |  |  |  |  |  |  |  |  | 100 |
| Rey-Galindo et al., (2020) | Y | Y | Y | Y | Y | Y | Y |  |  |  |  |  |  |  |  |  |  |  | 100 |
| Zhao et al., (2018) | Y | Y | Y | Y | Y | Y | Y |  |  |  |  |  |  |  |  |  |  |  | 100 |
| Kan‐Kilic et al., (2020) | Y | Y | Y | Y | Y | Y | Y | Y | N | Y | Y | Y | Y | Y | N | Y | Y | It is acknowledged that the sample is small, and the findings are not generalizable. Outliners and limitations are discussed. | 80 |
| Kan‐Kilic & Dogan, (2017) | Y | Y | Y | Y | Y | Y | Y | Y | N | Y | Y | Y | Y | Y | Y | N | Y | The authors acknowledge the limited sample size, which may constrain the generalizability of the findings. | 80 |
| Secchi et al., (2017) | Y | Y | Y | Y | Y | Y | Y | Y | Y | Y | C | Y | Y | Y | Y | Y | Y | The sample is representative in terms of age and VI types, but geographically limited to Florence. | 80 |
| Morag et al., (2016) | Y | Y | Y | Y | Y | Y | Y | Y | N | Y | Y | Y | Y | Y | Y | C | Y | The sample might not be representative due to the strategy used and limited number of people with each type of impairment. Only 2 people with VI were included. | 80 |
| Koutsoklenis & Papadopoulos, (2014) | Y | Y | Y | Y | Y | Y | Y | Y | N | Y | Y | Y | Y | Y | Y | C | Y | While the sample is relevant, it might not be representative of the target population. | 80 |
| Koutsoklenis & Papadopoulos, (2011) | Y | Y | Y | Y | Y | Y | Y | Y | N | Y | Y | Y | Y | Y | Y | Y | Y | All participants are recruited from one organization. The study does not explicitly consider factors such as age, gender, or degree of VI in the recruitment process. | 80 |
| Koutsoklenis & Papadopoulos, (2011a) | Y | Y | Y | Y | Y | Y | Y | Y | C | Y | Y | Y | Y | Y | Y | Y | Y | Not enough information is provided to determine if the sample is representative. All participants are recruited from one organization. | 80 |
| Thapar et al., (2004) | Y | Y | Y | Y | Y | Y | Y | Y | N | Y | Y | Y | Y | Y | Y | C | Y | The sample is very small and may not fully represent the diversity of people with MI and VI. | 80 |
| Cushley et al., (2023) | Y | Y | Y | Y | Y | Y | Y | Y | C | Y | N | Y | Y | Y | Y | N | Y | The information provided on sampling is insufficient. Using an online questionnaire for VI participants is prone to bias. | 60 |
| Nagassa et al., (2023) | Y | Y | Y | Y | Y | Y | Y | Y | N | Y | C | Y | C | Y | Y | C | Y | The small sample might not be representative. Not enough info is provided on potential bias. | 60 |
| Castle et al., (2022) | Y | Y | Y | Y | Y | Y | Y | Y | C | Y | C | Y | Y | Y | Y | N | Y | The study does not provide details on the survey's response rate or discuss the suitability of the sampling strategy. | 60 |
| Müller et al., (2022) | Y | Y |  |  |  |  |  | Y | N | Y | N | Y |  |  |  |  |  | The convenience sample might not be representative. The authors acknowledge a bias toward technophile participants as the survey was online. | 60 |
| Campisi et al., (2021) | Y | Y | Y | Y | Y | Y | Y | Y | C | Y | C | Y | Y | Y | Y | N | Y | Not enough information is provided to determine if the sample is representative of the broader population. Details of the response rate are not fully presented. | 60 |
| Gupta et al., (2020) | Y | Y | Y | Y | C | N | Y |  |  |  |  |  |  |  |  |  |  | Some disability groups are under-represented which limits the strengths of conclusions drawn. | 60 |
| Lukman et al., (2020) | Y | Y |  |  |  |  |  | Y | C | Y | C | Y |  |  |  |  |  | The paper does not provide details about eligibility criteria for recruitment and response rate. | 60 |
| Toyoda et al., (2020) | Y | Y | Y | Y | Y | Y | Y | C | N | Y | Y | Y | Y | Y | Y | C | Y | Information on the sampling and recruitment process is not sufficiently detailed. | 60 |
| Papadopoulos et al., (2018) | Y | Y |  |  |  |  |  | Y | N | Y | C | Y |  |  |  |  |  | Convenience sampling is used. Eligibility criteria and response rate data are not discussed. | 60 |
| Huang & Yu, (2013) | Y | Y | Y | Y | Y | Y | Y | Y | N | Y | C | Y | Y | Y | N | N | Y | The information provided on the sampling strategy is not sufficient. The small sample is claimed to be suitable for the chosen method. | 60 |
| Picard & Pry, (2009) | Y | Y |  |  |  |  |  | Y | N | Y | C | Y |  |  |  |  |  | While the sample is relevant, it does not fully represent the needs of the target population. | 60 |
| O. Belir, (2021) | Y | Y | Y | Y | Y | Y | Y | C | N | Y | N | Y | Y | Y | Y | N | N | The study uses a convenience sample of architecture students which is not representative of the VI population and poses bias. | 40 |
| Engel et al., (2020) | Y | Y |  |  |  |  |  | Y | N | Y | N | C |  |  |  |  |  | The majority of participants are from Germany, which may limit generalizability. The online format of the survey might exclude some VI users and pose bias. | 40 |
| Jeamwatthanachai et al., (2019b) | Y | Y | Y | Y | Y | Y | Y | Y | C | Y | C | C | Y | Y | Y | N | Y | The study does not explicitly describe the statistical analysis used. Divergencies are not discussed. | 40 |
| Korček & Rollova, (2014) | Y | Y | Y | Y | C | N | N |  |  |  |  |  |  |  |  |  |  | The methods used for data collection (audits) and analysis are not sufficiently detailed. So, findings cannot be assessed for accuracy. | 40 |
| Rousek & Hallbeck, (2011) | Y | Y | Y | N | Y | Y | Y | C | N | Y | C | Y | N | Y | Y | N | Y | There is not enough information on the eligibility criteria, sampling, or recruitment process. | 40 |
| Rousek & Hallbeck, (2011a) | Y | Y |  |  |  |  |  | N | N | Y | N | Y |  |  |  |  |  | While the sample (50 healthy university students wearing VI goggles) is relevant, it does not fully represent the needs of the target population. | 40 |
| Jeamwatthanachai et al., (2019a) | Y | Y | Y | N | Y | N | Y | N | N | Y | C | N | Y | N | N | N | Y | The BRS is tested with experts only which may not fully represent the needs of primary users. The links between Quan. and Qual. data are not discussed. | 20 |
| Синицын & Запорожец, (2021) | N | C |  |  |  |  |  |  |  |  |  |  |  |  |  |  |  | Research questions are not clear | N/A |
| Im & Sang, (2021) | N | C |  |  |  |  |  |  |  |  |  |  |  |  |  |  |  | Research questions are not clear | N/A |
| Swaminathan et al., (2021) | N | C |  |  |  |  |  |  |  |  |  |  |  |  |  |  |  | Research questions are not clear | N/A |
| Afshary et al., (2019) | N | C |  |  |  |  |  |  |  |  |  |  |  |  |  |  |  | Research questions are not clear | N/A |
| Belir, (2018) | N | C |  |  |  |  |  |  |  |  |  |  |  |  |  |  |  | Research questions are not clear | N/A |
| Omori et al., (2015) | N | C |  |  |  |  |  |  |  |  |  |  |  |  |  |  |  | Research questions are not clear | N/A |
| Tajgardoon & Karimi, (2015) | Y | Y |  |  |  |  |  | - | - | Y | - | - |  |  |  |  |  | While using a quantitative approach, this paper does not involve collecting data from human participants. So, most questions are not applicable. | N/A |
| Fixova et al., (2014) | N | C |  |  |  |  |  |  |  |  |  |  |  |  |  |  |  | Research questions are not clear | N/A |
| McIntyre & Hanson, (2014) | N | C |  |  |  |  |  |  |  |  |  |  |  |  |  |  |  | Research questions are not consistent and clear | N/A |
| O. Belir & Onder, (2013) | N | C |  |  |  |  |  |  |  |  |  |  |  |  |  |  |  | Research questions are not consistent and clear | N/A |
